# Squamous cell carcinoma in chronic osteomyelitis: a case report and review of the literature

**DOI:** 10.1186/s13256-016-1002-8

**Published:** 2016-08-04

**Authors:** Gaetano Caruso, Emanuele Gerace, Vincenzo Lorusso, Rosario Cultrera, Loredana Moretti, Leo Massari

**Affiliations:** 1 Orthopaedic and Traumatology Unit, Azienda Ospedaliero Universitaria di Ferrara Arcispedale Sant’Anna, University of Ferrara, Via Aldo Moro 8, 44124 Ferrara, Italy; 2 Infectious Diseases Unit, Azienda Ospedaliero Universitaria di Ferrara Arcispedale Sant’Anna, University of Ferrara, Via Aldo Moro 8, 44124 Ferrara, Italy; 3 Plastic and Reconstructive Surgery, Azienda Ospedaliero Universitaria di Ferrara Arcispedale Sant’Anna, Via Aldo Moro 8, 44124 Ferrara, Italy

**Keywords:** Osteomyelitis, Squamous cell carcinoma, Tibia, Amputation, Bioglass, Chronic wound, Exposed fracture

## Abstract

**Background:**

Chronic osteomyelitis is a challenging problem, and malignant transformation is a rare occurrence. We report a case of a patient with squamous cell carcinoma arising from an osteomyelitic hotbed and discuss through a literature review the etiopathogenesis, diagnosis, and treatment of this lesion.

**Case presentation:**

A 69-year-old Italian man had sustained an exposed tibial fracture 40 years ago during a road accident, for which he had undergone various surgical osteosynthesis treatments with multiple antibiotic therapies. He presented to our hospital because of recurrence of a fistula at the proximal third of the anterior region of the tibia. For 2 months, we treated the lesion with antibiotics, and local medication with curettage. We saw no evidence of lesion improvement, and we advised the patient to undergo a knee amputation, which he refused. The alternative we chose was a surgical toilet of the osteomyelitic hotbed and used bioglass as a bone substitute. After 2 months of follow-up, we noticed a fulminating, budding formation in the area of the surgical wound that turned out to be a squamous cell carcinoma on biopsy. The patient again refused the amputation and underwent a wide-margin surgical debridement. After 2 months, the carcinoma recurred, and an above-the-knee amputation was performed.

**Conclusions:**

Our experience with this case indicates that amputation is the most appropriate treatment for squamous carcinoma occurring in patients with chronic osteomyelitis. To avoid risks of lymphonodular and organ metastasization, this radical surgical procedure should not be delayed. Early diagnosis and timely therapy can prevent amputation only in selected cases. Surgeons who treat osteomyelitis and chronic wounds should be aware of the risk of tumor degeneration. Squamous cell carcinoma associated with chronic osteomyelitis has a low-grade malignancy, but implications of lymphonodular involvement and organ metastasis should not be excluded.

## Background

Chronic osteomyelitis is considered a great challenge for the orthopedist. The characteristic bone histology and the ability of bacteria to adapt to this particular microenvironment make these infections among the most insidious in the human body. Osteomyelitis is an inflammation involving the osteoarticular apparatus and its medullary canal that evolves in a progressive destruction of bone tissue. The characteristics of this phenomenon are bacterial colonies organized and protected by biofilm, a polymeric matrix the colonies excrete, often a polysaccharide. It is hard to breach this barrier, and antibiotics and disinfectants do their best to kill these shielded bacterial colonies. Furthermore, bacterial colonies enter a semidormant state that stretches pharmacodynamic effects of chemotherapies and the immune system, both specific and nonspecific. In this report, we present a case of recurrent squamous cell carcinoma in a patient with chronic osteomyelitis for which surgical amputation of the limb was initially avoided.

## Case presentation

**Fig. 1:**
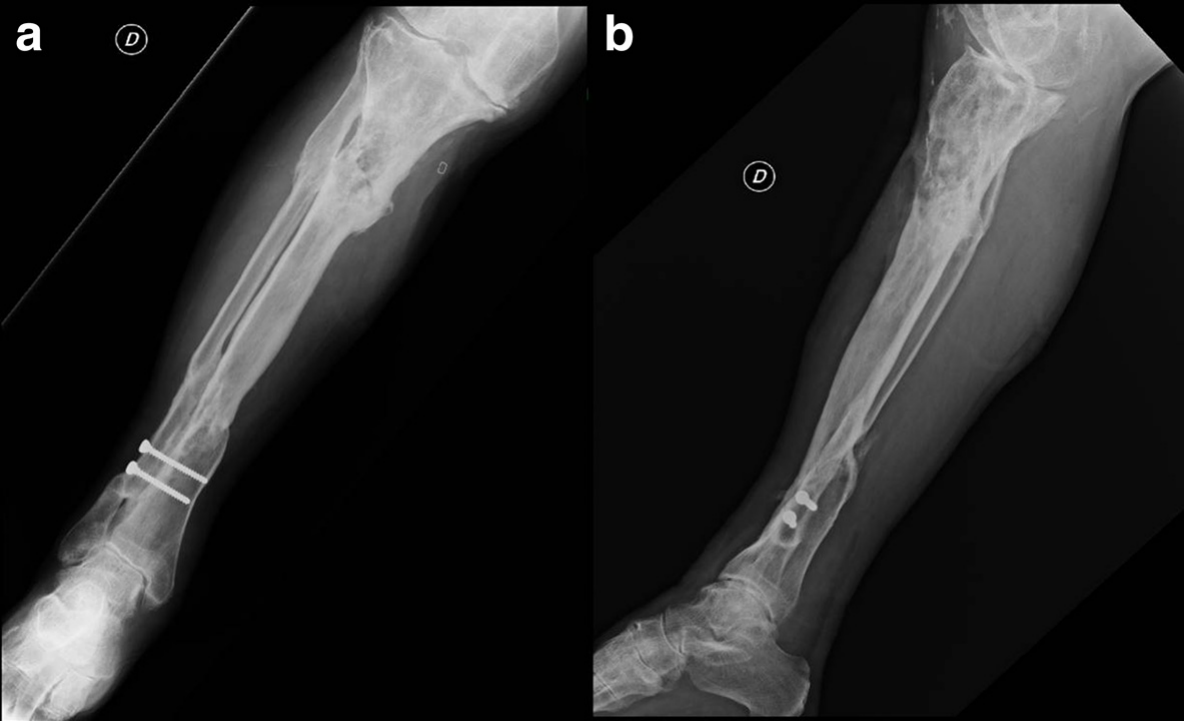
Anteroposterior (**a**) and lateral (**b**) x-rays of the tibia showing an area of inhomogeneous osteostructural depletion at the proximal third of the tibia

A 69-year-old Italian man came to our hospital for treatment of a 5-mm fistula at the proximal third of the anterior region of the tibia. He was affected by chronic osteomyelitis because of an exposed fracture that had occurred 40 years ago. Plain x-rays showed an osteolytic area above the wound (Fig. 1). In the first years following the trauma, he had undergone many osteosynthesis surgical interventions and antibiotic regimens. The patient was self-sufficient, in good clinical condition, and had no comorbidities. The neurovascular status of his affected leg was intact. In 1994, he had undergone a bilateral inguinal herniotomy, and in 2000 he had had a hemorrhoidectomy. He contracted hepatitis C virus during his first hospitalization after the trauma.

**Fig. 2:**
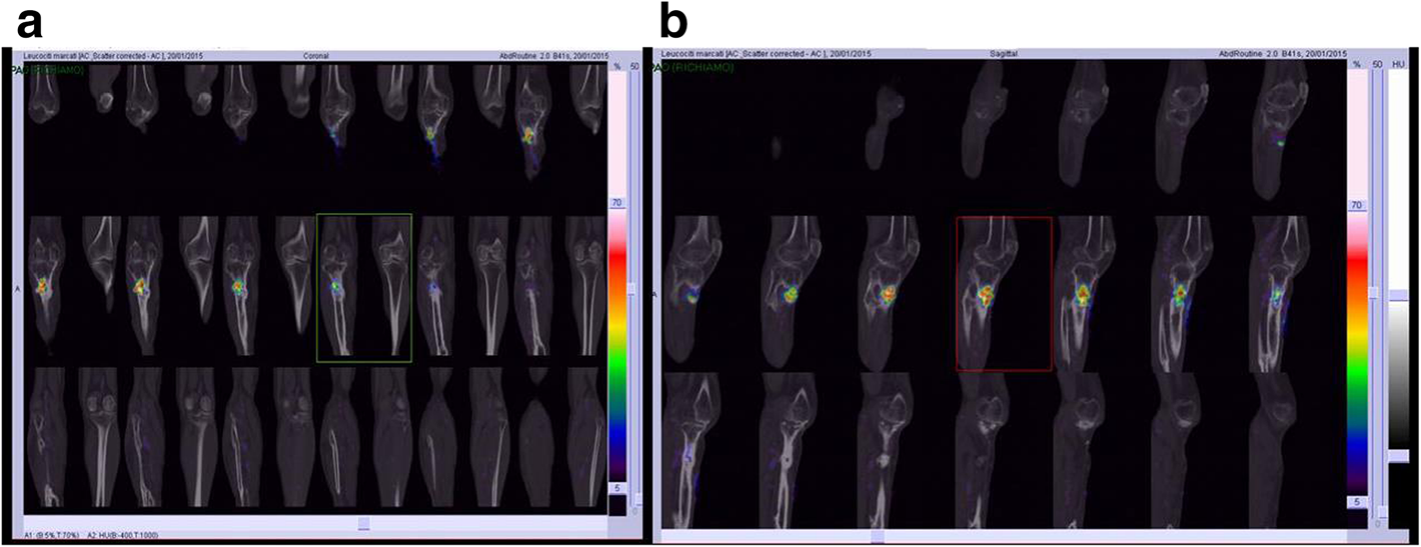
Scintigraphic (**a** and **b**) of the tibia presenting a polylobed area with an increased distribution of radiocytes in the proximal third of the tibia that involves both the bone and the soft tissues

**Fig. 3:**
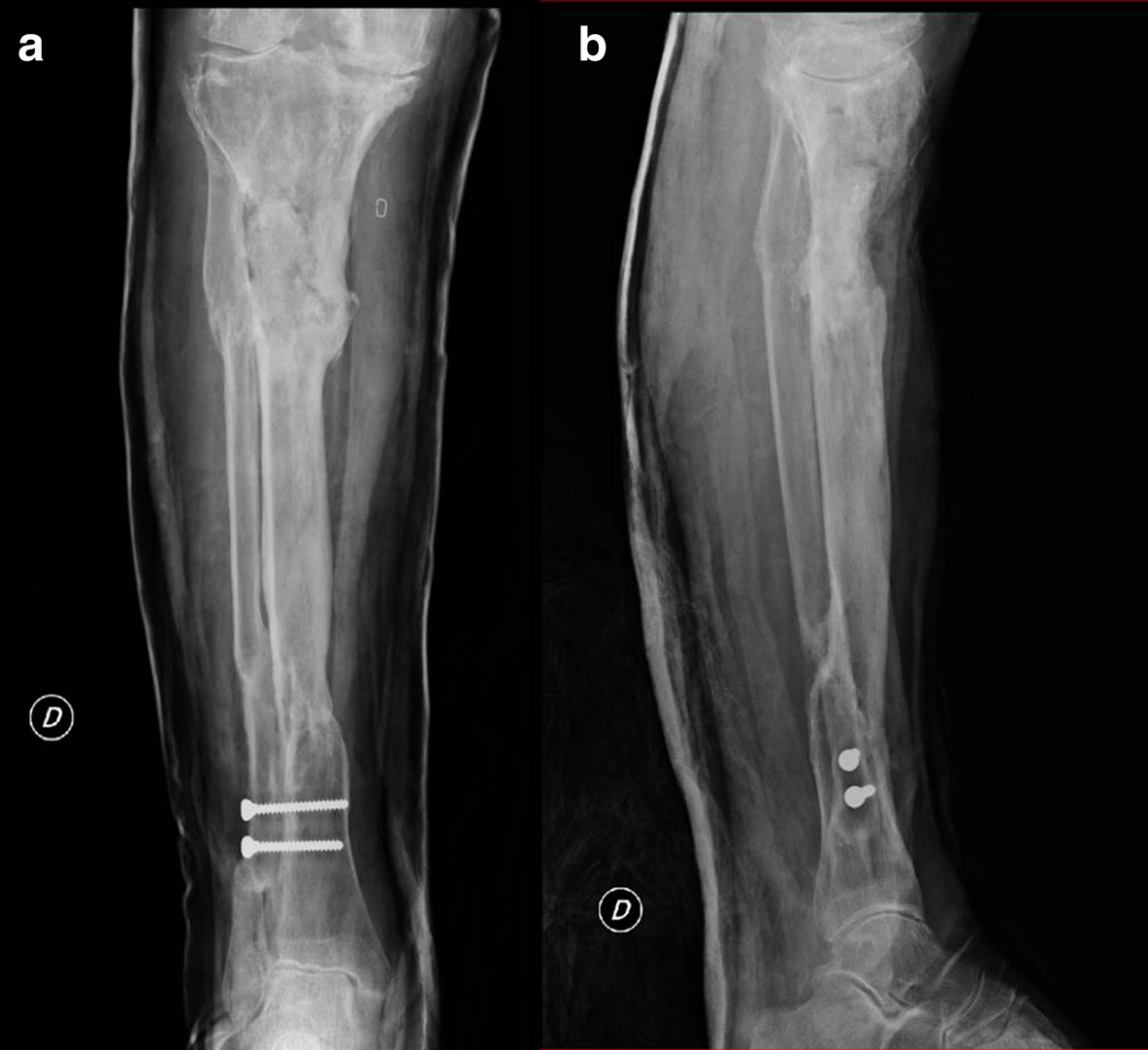
Anteroposterior (**a**) and lateral (**b**) x-rays obtained after the first surgical intervention, in which bioglass was applied

**Fig. 4:**
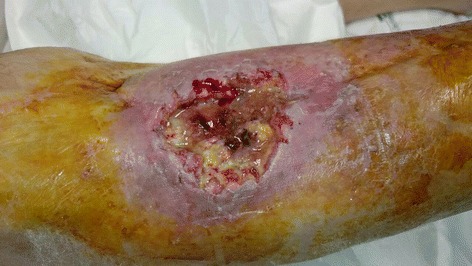
Clinical photography of the wound 2 months after the first surgical intervention, showing the squamous cell carcinoma

**Fig. 5:**
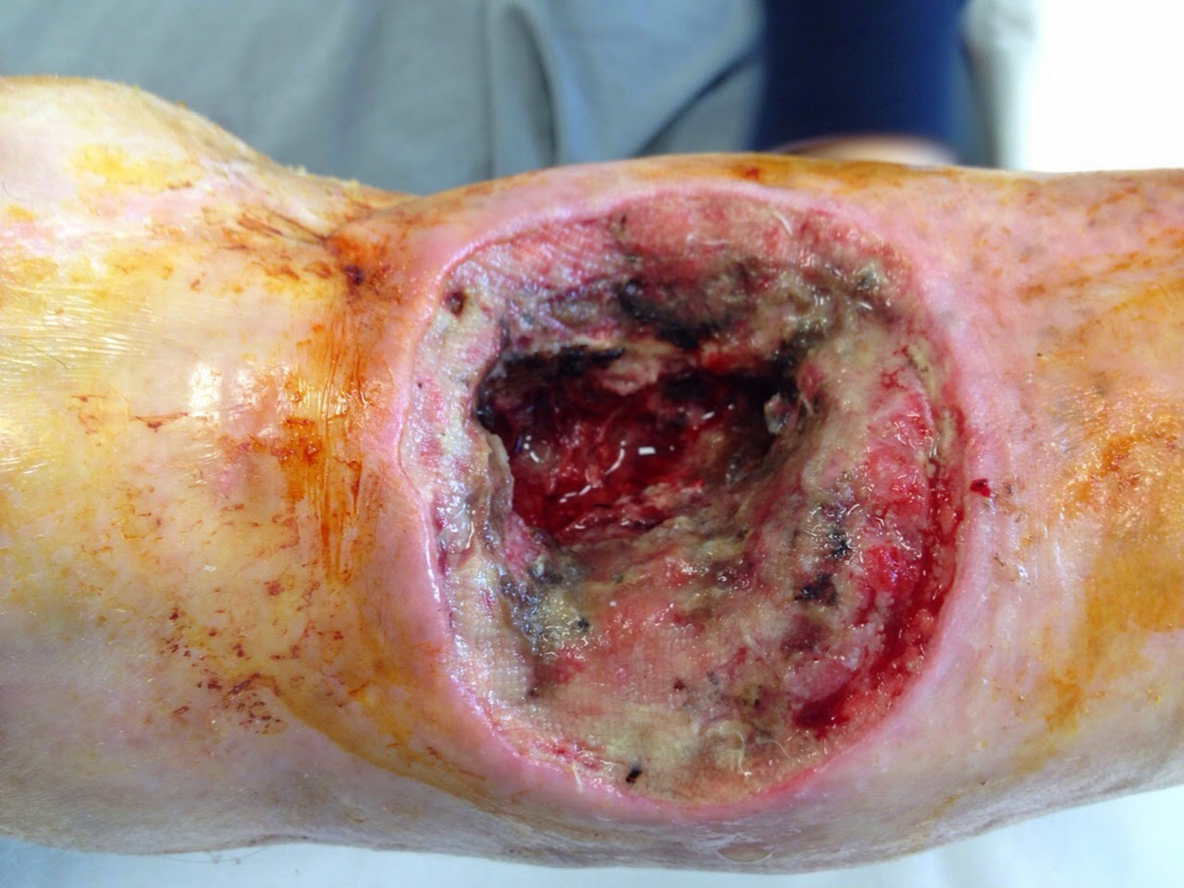
Clinical photography of the wound after wide-margin surgical debridement and bioglass removal

**Fig. 6:**
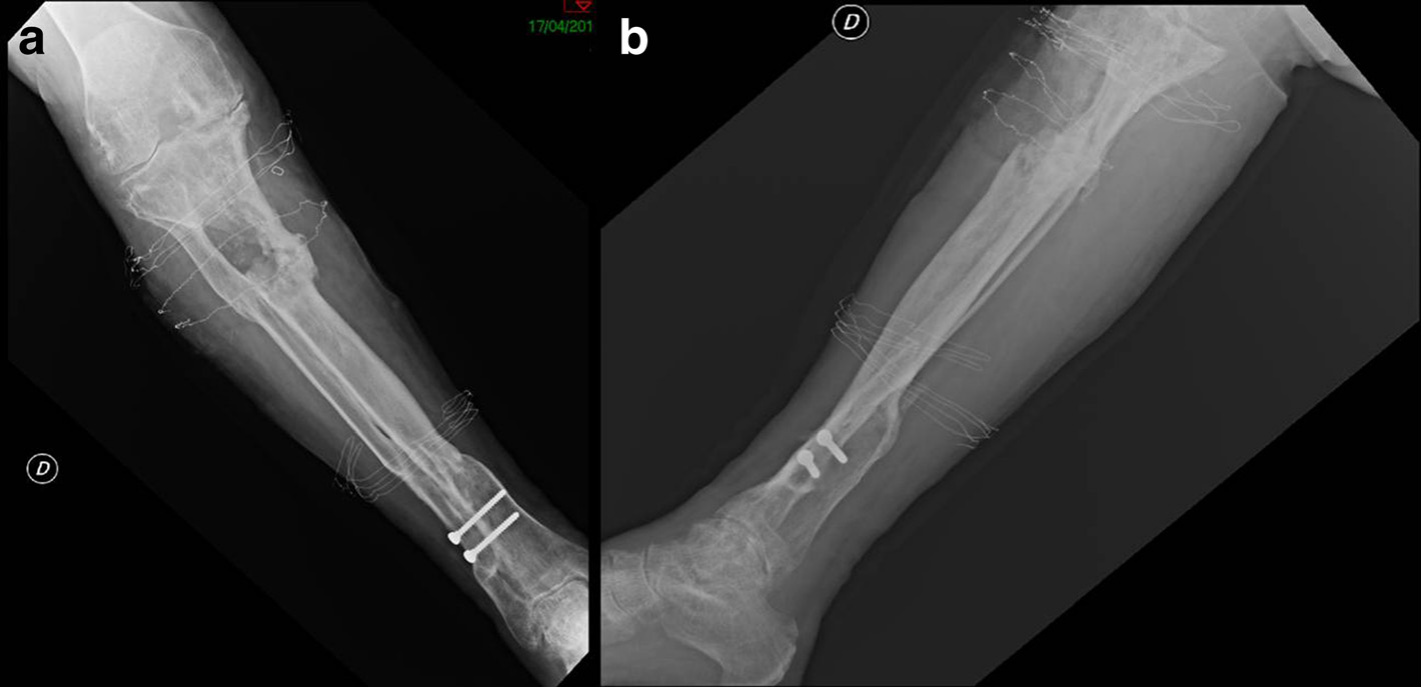
Anteroposterior (**a**) and lateral (**b**) x-ray demonstrating the bone depletion after the second surgical procedure

**Fig. 7:**
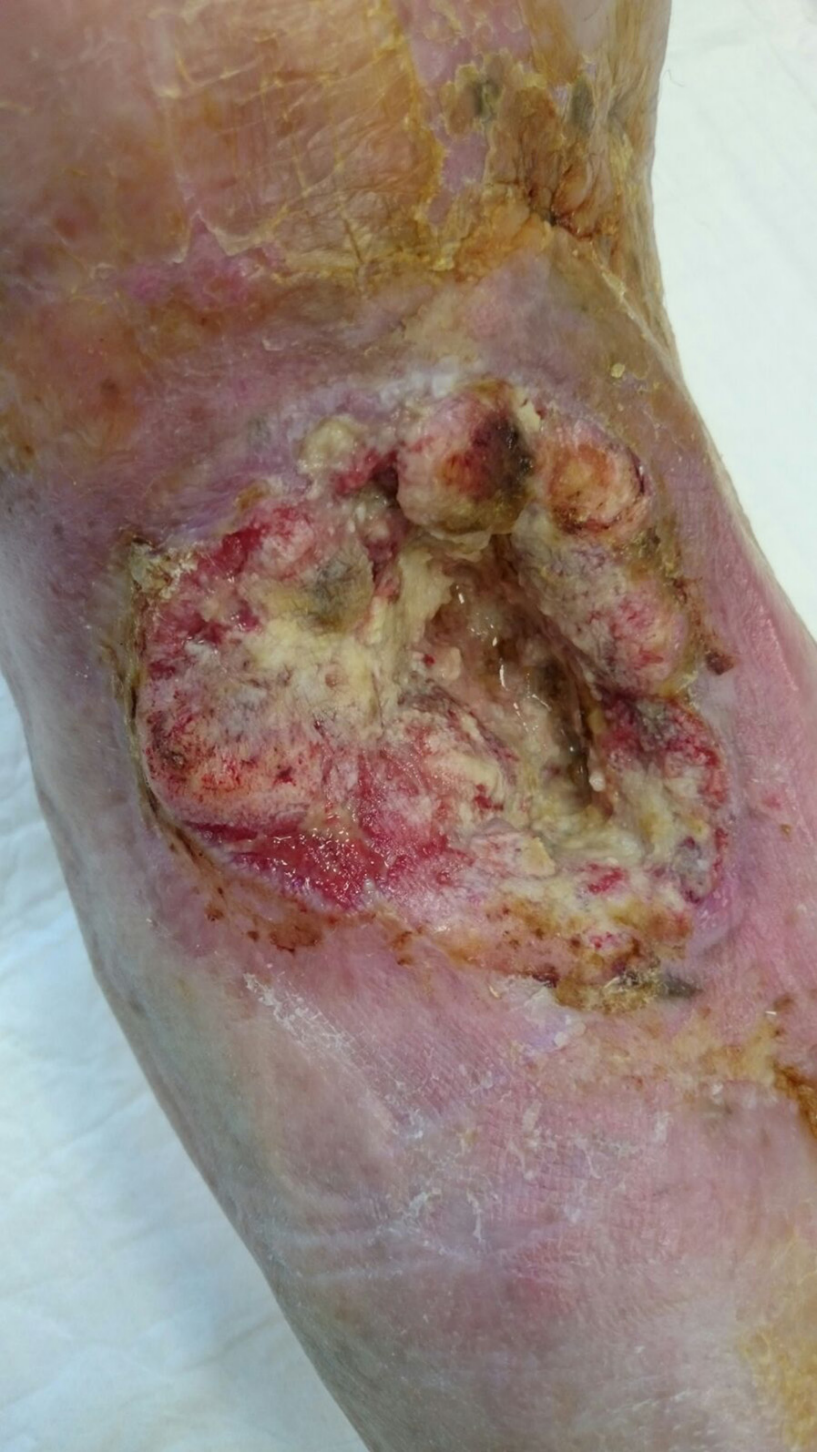
Clinical photography of the tibia demonstrating the squamous cell carcinoma recurrence

Scintigraphy confirmed the presence of an intraosseous infective process in the involved region (Fig. 2). A swab culture of the fistula was positive for *Bacteroides fragilis* and *Corynebacterium striatum*, so the patient underwent therapy with metronidazole (500 mg by mouth every 6 h). After 30 days, the fistula had increased in size, and fibrotic tissue, perilesional erythema, and malodor were present. We recommended to the patient that he undergo amputation of the knee, a treatment that he refused. Therefore, he underwent a surgical toilet of the osteomyelitic hotbed, and inflammatory tissue and macerated bone were removed. The bone defect was treated with bioglass (Fig. 3). The surgical wound was closed without any skin grafting. The result of an intrasurgical swab culture was positive for *Pseudomonas aeruginosa*. We started the patient on a multiple-antibiotic regimen with amikacin (500 mg intravenously every 12 h), meropenem (2 g intravenously every 8 h), and ciprofloxacin (500 mg by mouth every 12 h). After 2 months of inpatient care, a fulminating budding formation was observed on the wound margins. A biopsy revealed that it was a squamous cell carcinoma (Fig. 4). The patient underwent whole-body computed tomography (CT), which revealed bone lesions and soft tissue masses. His neoplasia turned out to be T3N0M0; according to the American Cancer Society guidelines, it was at stage III [1]. He underwent a wide-margin surgical debridement and bioglass removal (Figs. 5 and 6). After 2 months, the malignant lesion recurred locally (Fig. 7), and we performed a below-the-knee amputation.

## Discussion

Neoplasms as a result of chronic insults were described as early as during the Roman Empire. Aulus Cornelius Celsius told of tumoral neoformations in correspondence to chronic wounds [2]. During the mid-19th century, Hawkins and Marjolin correlated these skin diseases with osteomyelitis [3]. In a Mayo Clinic study in which investigators analyzed about 4000 cases of chronic osteomyelitis, malignant lesions were noted in 23 % of the patients [4]. The incidence of skin lesions ranges between 0.2 % and 1.7 % of all patients affected by osteomyelitis [5, 6]. In developing countries, the percentages increase with delayed diagnosis and inadequate treatments [7]. Nowadays, trauma is the most common cause of osteomyelitis [5]: Infection rates range from 4 % to 64 % in open long bone fractures, and the incidence is increasing [8]. The occurrence of hematogenous osteomyelitis is declining, with the incidence dropping from 87 to 42 cases per 10,000 [8]. The duration of osteomyelitis appears to be the principal factor related to carcinogenesis onset, with a minimum latency period of 20 years or more [9]. Males are affected more often than women, with a predominance of 85 %, and patients’ are typically aged between 50 and 60 years old [5]. The tibia, femur, and foot are the most frequent locations [5, 10]. Clinical signs that should alert the clinician about malignant transformation include increased pain, blood, or foul release from the sinus; progressive bone destruction and erosion; and a growing mass in the area of the wound [2]. To prevent local invasion and metastatic spread, treatment should not be delayed. The pathogenesis of this neoformation is still under debate, but the most widely accepted theory is focused on the chronic inflammatory state [7, 11]. In these conditions, the immune system is dysregulated: Inflammatory mediators and cytokines expressed by the immune system modulate the genic expression of various proteins, including p53 [11]. Furthermore, avascular areas and lymphatic duct obliteration [7] are conditions that discourage antigen presentation. Polymicrobial infection sites are also characterized by horizontal gene transfer and consequent latent mutations that interfere with the immune response [12]. There is evidence that carcinomatous transformation can follow a shift in bacterial flora. Gram-positive flora can be replaced by predominant gram-negative flora that produce endotoxins associated with cancer [12].

Squamous cell carcinoma is characterized by an intraepidermal proliferation of atypical keratinocytes [13], when associated with chronic osteomyelitis is usually of low grade malignancy [14].

The diagnosis can be made with the use of a primary and secondary imaging medical device. Magnetic resonance imaging can be useful to differentiate squamous cell carcinoma from other soft tissue neoplasms [15]. Whole-body positron emission tomography-CT can clarify a suspicion of metastasis, especially in the lungs, the most common site. Shave biopsy, incisional biopsy, excisional biopsy, and punch biopsy are the settling examinations [14]. They should include portions of the entire lesion: ulcer, sinus, and the marrow space.

Patients affected by chronic osteomyelitis with recurrent exacerbations undergo frequent hospital admissions, pharmacological therapies, and surgical procedures during their lifetimes. Several authors have suggested that amputation is the definitive treatment [5, 10, 16, 17]. Body image anxiety, social discomfort, and depression are frequent consequences of lower limb amputation [18, 19]. It is a tough choice that involves both physical and psychological issues, but in our experience it can guarantee better quality of life. In some selected cases and in the absence of metastasis, it is possible to evaluate a wide excision of the lesion, making use of Mohs micrographic surgery to resect at least 2 cm of tumor-free margins. Our patient at first did not accept the amputation. In his opinion, it would have prevented him from riding motorbikes, his preferred sport activity, and it would have altered his social role. Different authors have reported that persons with lower limb amputation can restore patients’ life habits [19] and riding and/or driving capabilities [20], but barriers such as family understanding, inadequate legislation, and confusion over various driving adaptations can slow the return to the community [19] and to the activities conducted before the amputation [20]. Our patient underwent a toilet of the osteomyelitic hotbed. The bone substitute we used is BonAlive S53P4 bioglass (BonAlive Biomaterials, Turku, Finland). Bone graft substitutes are widely used in orthopaedics since several decades with good outcomes whenever there is a need of a matrix capable to bridge regenerative or reparative biological effects. In most cases bone substitutes have just an osteoconductive effect and their efficacy is restricted only to certain conditions [21, 22]. Autologous or heterologous bone transplants, in addition to being a substitute, have an osteostimulative effect. Unfortunately, the autologous effects are related to the adverse effects and comorbidities associated with a second surgical procedure, while the heterologous effects are related to infective risks and immune intolerance. Bioglass is a material with both osteoconductive and osteostimulative effects, as well as having antibacterial properties [21, 22].

## Conclusions

Malignant transformation in chronic osteomyelitis is a rare but unfavorable condition. Early diagnosis on the basis of information related to both clinical signs and imaging can lead to less invasive surgical treatments. Amputation is the definitive treatment of recurrent chronic osteomyelitis even in the absence of neoplastic transformation. It should not be delayed, and the patient’s compliance is of prime importance. Surgeons should be aware of the risk of tumor degeneration in patients with osteomyelitis and chronic wounds.

## Abbreviation

CT, computed tomography
